# Virtual patient design: exploring what works and why. A grounded theory study

**DOI:** 10.1111/medu.12151

**Published:** 2013-05-12

**Authors:** James Bateman, Maggie Allen, Dipti Samani, Jane Kidd, David Davies

**Affiliations:** 1 Department of Medical Education Research and Development, Warwick Medical School, University of WarwickWarwick, UK; 2 Medical Education Department, University Hospitals Coventry and Warwickshire National Health Service (NHS) TrustCoventry, UK

## Abstract

**Objectives:**

Virtual patients (VPs) are online representations of clinical cases used in medical education. Widely adopted, they are well placed to teach clinical reasoning skills. International technology standards mean VPs can be created, shared and repurposed between institutions. A systematic review has highlighted the lack of evidence to support which of the numerous VP designs may be effective, and why. We set out to research the influence of VP design on medical undergraduates.

**Methods:**

This is a grounded theory study into the influence of VP design on undergraduate medical students. Following a review of the literature and publicly available VP cases, we identified important design properties. We integrated them into two substantial VPs produced for this research. Using purposeful iterative sampling, 46 medical undergraduates were recruited to participate in six focus groups. Participants completed both VPs, an evaluation and a 1-hour focus group discussion. These were digitally recorded, transcribed and analysed using grounded theory, supported by computer-assisted analysis. Following open, axial and selective coding, we produced a theoretical model describing how students learn from VPs.

**Results:**

We identified a central core phenomenon designated ‘learning from the VP’. This had four categories: *VP Construction*; *External Preconditions*; *Student–VP Interaction*, and *Consequences*. From these, we constructed a three-layer model describing the interactions of students with VPs. The inner layer consists of the student's cognitive and behavioural preconditions prior to sitting a case. The middle layer considers the VP as an ‘encoded object’, an e-learning artefact and as a ‘constructed activity’, with associated pedagogic and organisational elements. The outer layer describes cognitive and behavioural change.

**Conclusions:**

This is the first grounded theory study to explore VP design. This original research has produced a model which enhances understanding of how and why the delivery and design of VPs influence learning. The model may be of practical use to authors, institutions and researchers.

## Introduction

Virtual patients (VPs) are computerised representations of realistic clinical cases.^1^ It has been suggested that VPs are best applied to teach clinical reasoning skills.^2^ Analogous to that on other web-based educational interventions, much of the focus on VP adoption has concerned ‘if’ rather than ‘how’ they should be designed and used.^3^,^4^ Recent advances in technology and internationally adopted technical standards have potentially changed the definition of what a VP is as VPs can now be shared, edited and repurposed between institutions.^5^,^6^ Virtual patients have the potential to deliver education to large numbers of students at a relatively low cost, which will be important in addressing the challenges that will face medical education in coming decades.^7^,^8^ Existing principles for multimedia instructional design have been established^9^,^10^ and studied in different instructional formats,^11^ including preliminary work in VPs. For example, researchers have compared the delivery of text alone against that of text and supporting images.^12^ Other general instructional design principles, such as those implied by cognitive load theory, are valid for some formats, including lecturing and e-learning.^13^ However, such theory cannot be applied logically to VPs because they are often used to teach realistic scenarios in which the cognitive load is intentionally heavy in order to replicate that in the clinical scenario.

Given the wide range of VP design typologies, educational theory alone has limited ability to predict their success and the interplay among them.^14^ The optimal design of VPs has therefore emerged as the subject of an important research question in the literature and has not been adequately addressed.^6^ Qualitative methodologies including grounded theory provide opportunities to analyse, identify and explore experiences, and thus understand how and why different VP design properties support learning.

### Objectives

This study has a principal aim and a subsidiary aim. The principle aim is to identify and explain how the design properties thought to be important in VPs influence medical student interaction with VPs. The subsidiary aim is to gain insight into which of the numerous VP design properties would be particularly relevant to study in future research.

## Methods

### Study design

This is a grounded theory focus group study in which medical undergraduates from one medical school evaluate two substantial VP cases authored specifically for this research. We use one school of grounded theory, the ‘classic grounded theory’ approach proposed by Juliet Corbin and Anselm Strauss,^15^,^16^ which uses iterative sampling, conceptual memoing, and the simultaneous collection and analysis of data. We consider our epistemological stance as analogous to that of Corbin.^16^ We acknowledge the positivist origins of grounded theory, whilst recognising and valuing constructivism and reflexivity in the context of our pragmatist theoretical orientation and training.^16^ The purpose of using grounded theory is to develop a theoretical explanation of ‘what is going on’, which, in this instance, refers to how and why design properties influence the effectiveness of VPs, by evaluating participant accounts and descriptions of their experiences. Our research protocol and research materials were subject to external peer review and were subsequently granted institutional ethics committee approval.

### Virtual patient authoring

We authored two 30-minute VP cases, each of which intentionally included a number of design variables. We selected design features within the research group by consensus following a literature review of VPs and a review of publicly accessible VP cases. We chose only design features compatible with international VP interoperability standards produced by Medbiquitous.^17^ We used the computer software DecisionSim Version 2.0 (Decision Simulation LLC, Pittsburgh, PA, USA) to author and deliver the cases following a technology appraisal of authoring tools. A detailed description and pictorial representation of our VP design variables can be found in supplementary material Table S1 and Fig. S1 (online). These variables included: branching and linear case narratives; freedom of navigation through the case; visible scoring systems; different question types such as multiple-choice questions; key feature problems, and Bayesian reasoning. Different learning strategies included: worked examples; listing of differential diagnoses; explicit identification of supporting information for and against particular diagnoses; information presentation techniques such as authentic clinical letters or salient results; post-case resources; data-gathering techniques (menu-driven history taking), and the provision of different feedback mechanisms with explicit and tacit feedback. These variables have been explicitly highlighted as important in both the VP and simulation literature.^2^,^6^,^14^,^18^,^19^

We integrated these designs into two substantial 30-minute VP cases focusing on core musculoskeletal medicine disease presentation. The cases were authored specifically for this research by an author experienced in producing several cases for a previous large VP research project.^20^ We piloted the cases with two hospital consultant doctors, two general practitioners and two doctors in medical specialist training. This allowed us to correct grammatical, presentation and technical errors, judge the duration of the cases, and check item difficulty.

### Participants and sampling

Participants were students in Years 2 and 4 at a graduate-entry medical school, which runs a 4-year programme. All were on placements at a university teaching hospital, which utilises VPs in some teaching modules. We used an iterative purposeful sampling technique to recruit volunteers.^21^,^22^ Recruitment was voluntary; students were told the subject was musculoskeletal education research, but were blind to the use of e-learning. Students were given participant information sheets and asked to sign informed consent papers. There was no financial incentive to participate. We authored and piloted a funnelled questioning route using established focus group methodology, which we had piloted for acceptability, usability and question clarity.^23^ This can be seen in Appendix S1 (online). We invited up to eight student participants to each focus group. We conducted six focus groups with a total of 46 participants. We used saturation sampling to decide when to terminate sampling as part of the constant comparative data collection and analysis.^21^ As no new themes emerged during the sixth focus group, data collection was halted.

### Data collection and analysis

Participating students completed the two VPs using a unique online identifier; after each case, students completed a self-report VP evaluation using an established VP evaluation instrument.^24^ Data logs of student decisions and evaluations were recorded as additional sources of data to help inform the grounded theory analysis. They were explicitly not included in the protocol as subject to detailed statistical analysis for reasons of study design, sample size, power and sampling techniques. Following the evaluation, students took part in a 1-hour focus group discussion conducted according to an established methodology, with a pre-planned written funnelled questioning route. We used an experienced focus group facilitator (JB) and moderator (DS), who made field notes during the sessions. Focus groups were digitally recorded. The principal researcher transcribed, re-read and coded the interviews to develop a preliminary open coding structure, which was reviewed by other members of the research team. In line with the grounded theory approach, data analysis proceeded at the end of the focus group, prior to participant sampling for the next focus group. We used the computer-assisted qualitative data analysis software (caqdas) NVivo Version 9.0 (QSR International Pty Ltd, Doncaster, Vic, Australia) to facilitate analysis, interrogate the dataset, memo and provide an audit trail within the research team.^25^ The research team included a medical educationalist, a university hospital head of medical education and a doctoral student. In our initial open coding we used a line-by-line approach, labelling phenomena using descriptive codes, and direct quotations from students. The latter are known by convention as *in vivo* codes.^26^ We then proceeded to ‘axial coding’, a grounded theory method whereby codes are connected and grouped by virtue of common properties and dimensions.^15^ We used scheduled regular research group meetings to describe, refine, review and seek alternative explanations for phenomena observed. Emergent themes guided sampling. For example, as student clinical experience emerged as an important theme, we purposefully sampled more junior students with little subject-specific experience in focus groups 5 and 6. Finally, in selective coding we identified a central core category which could be linked to all other categories using a paradigm described by the school of grounded theory.^15^ Through a series of iterations and discussions within both the research group and institution educational board meetings, we then abstracted and verified a pictorial model describing how an individual student learns from a VP.^27^

## Results

Of the 48 students who volunteered to participate, 46 attended (19 males and 27 females). Of these, 31 students were in Year 4 and 15 were in Year 2. All students completed the VPs, evaluations and focus group discussion.

From the analysis theory we identified a core phenomenon, ‘learning from the VP’, to which four main categories related. As might be expected, these categories included *VP Construction*, which refers to material that an author integrates into a case; *External Preconditions*, which refers to student- and institution-centred factors; *Student–VP Interaction*, which refers to the fluctuating interface between the student and the VP, and *VP Consequences*, which refers to the after-effects on the student, which are co-dependent on the three prior categories. Examples of each category are given in Tables 1–3.

**Table 1:** The first two categories, ‘VP construction’ and ‘External preconditions’ of the central phenomenon ‘learning from the VP’. *In vivo* codes (direct quotes) are in italics with quotation marks

Category 1. VP construction: The VP properties that are designed into a case
**Clinical properties. Clinical properties authored into the virtual patient by an author**
“**Real Life**”: The clinical properties authored into a VP case and how they relate to actual clinical practice
Environment: Simulation of the clinical environment, for example GP having past health care records from a patient
Authenticity: The authenticity of the narrative and supporting educational materials in the case
“I like the way it's based on the way we've been taught so far… you start with the history and you take a detailed history, and I like that it actually gave you the option of collecting that history from that patient. … it still followed the steps that you would take in a normal situation which is getting a clear history, a systems review included of a patient and a condition… definitely something that applies to real life and definitely something that would be useful.” EA, FG6, Year 2 student
Scope and content: The extent to which health care domains are explored by the case, such as clinical knowledge, professionalism, clinical reasoning, local health care policy, and health service structure
**Pathway Flux:** How the flow of clinical and other information is presented between the student and the VP
Channels and dams: The degree of freedom given to the student over their actions, progression and the narrative in the case
Evolution-Evaluation: The extent to which data and information is presented, reviewed and evaluated as the case progresses
Clinical Inertia: How case progression is resisted by the quantity, quality, completeness and relevance of pathways, data and activities that contribute to cognitive load, realism and difficulty
“the referral letter was good and bad, good because it's probably what we'd get, and bad because it was a bad referral letter… one of the questions was what is pertinent to this referral letter… and it had duration of symptoms, and you don't know how long its been going on for…” SR, FG3, Year 4 student
**Pedagogic properties: Teaching elements of VPs integrated into VP cases**
**Feedback**: How feedback is delivered to the students as they complete a VP case
Format effects: Implications of different formats such as a letter, or a phone call, at different times through the case
Tailoring: Extent to which student feedback is individualised, including comparisons with peer performance
Prompting reasoning: Approaches that explicitly drive structured clinical reasoning
“It was good to kind of think about the differentials… I do think the lack of knowledge was an influential factor, but it did help me question why is it that I'm including this one, and why is it that I'm including that one, I looked back to the history… you come across important factors… is that a long term condition, or is this acute… rule things out… I thought it was really good.” RR, FG5
**Decision Flux**: How decisions contribute to freedom to make decisions both correct and incorrect, and experience consequences of them.
**Consequence effect:** Extent to which students feel their decisions impact further down the case narrative.
**Limits and Forcing**: Being forced to undertake a particular action, decision, cognitive process or clinical experience irrespective of the apparent choices given
“I quite liked the way that sometimes they got you to pick only three questions, which kind of got you maybe to think rather than ask just random questions. Think where your thoughts were going and what questions were important” CD. FG6
**E-properties: Electronic properties used provoking comment and outside of normal expectations for electronic interfaces**
**E-Signposting**: The helpful effect of signposting students using images of locations and particularly patients
“I really liked on the first case the pictures. I know, I know it was just random adjudicators, but it kind of made you smile and if you've got that kind of visual stimulation, oh that's the GP OK, it kind of motivates you…” AR, FG1
**E-inertia**: Electronic properties authored into cases which produce slow or hinder a student interacting with a case.
**Non e-tasks**: The use of items that don't require actions by the student for example summarising elements on paper
**Software limits**: Desired software features from students, not present, which limit interaction.
“**Scroll scroll scroll**”: Impact of multimedia design including text format, length, steps, and image representation
“I think some of the pages were quite wordy, maybe it can be broken down into two instead of one, and squeezing all of the information into one page, it just gives me a headache” SS, FG2
**E-Error**: Electronic error as students sit a case, the cause of which may or may not be under the control of the case author

**Category 2. External preconditions: factors, external to the VP construction which influences its utility**
**Student centred: Student centred factors that influences the utility of the VP case**
**Electronic Prejudice**: How both prior positive and negative e-learning experiences prejudice the approach to a VP
**Global experience**: The global knowledge and skills a student possesses about medical problems and health care systems
**Student Goals**: The students personal goals for the learning activity, for example relating to assessment or professional development.
“I can see why its on there, because for finals, they are going to say, “what are your differentials, summarise the case in a sentence” AR, FG1, Year 4 student
**Organisation elements: An organisation's educational aims, curriculum, assessment and evaluation of students**
**Institution fingerprint**: The organisational style and expectations of students when approaching educational/assessment “I don't know how that reflects on our teaching… we're quite often lead to a single best answer” KG, FG1, Year 4 student
**Assessed curriculum**: Factors that relate to the pedagogic, assessment and curricular approaches of an educational institution
**Environmental elements**: Local factors (location, computer hardware) that influence how a student interacts with a VP case
“I know we had an hour, and at the end people were leaving so I felt, sort of, hurry up.” DE, FG3, Year 4 student

GP = general practitioner; FG = focus group

**Table 2:** Student–Virtual Patient (VP) Interaction, the third category from the central phenomenon ‘learning from the VP’. *In vivo* codes (direct quotes) are in italics with quotation marks

Category 3. Student–VP Interaction: the interaction between a student and a VP as the student completes a case
**Skipping threshold: a threshold above which negative behaviour patterns occur and interaction ceases to be constructive to learning**
**Efficient skipping**: engagement is limited by a drive to efficiently pick out activities that are perceived to add value or be important JC: ‘Some of the pages had a lot of words on, and my eyes go, nah there's a lot to read there, and there's nothing to input, and I don't have to give anything, so therefore there's no need for me to read it because it wasn't about the case. That's just me being lazy… AA: ‘I think by nature we're all quite lazy, and we'll be like “Nah”’JC: ‘Efficient’ JC and AA, Year 4 students, FG2
**Judged credibility**: a constant appraisal of the usefulness, quality and interactivity of the VP to judge whether to continue with the case
**Style:** approaches in which the style of questioning promoted lack of engagement and skipping ‘There were some of the questions where it was you selected one answer, and it wouldn't allow you to go through until you clicked the right one… At that point I'd given up and was just guessing, which obviously I would never do with a real patient'DG, Year 2 student, FG5
**Mental case building: interactions which help the student to construct a mental representation of the case**
**Learning and assessment focus**: added engagement resulting from perceived benefits in learning, future assessments or the workplace
‘**Thinking outside the list’**: strategies which encourage students to think outside a predetermined list of answers in the task
**Pathway growth**: the extent to which both decisions and branching pathways enhance students’ experiences, and experiential learning ‘I think I quite like the branching bit… because obviously in life there are a lot of different routes you can take and it doesn't necessarily mean one is… the best … I think it's good to go a little bit off track’JC, Year 4 student, FG2
**Mental case fracturing: interactions which impair the student's construction of a mental representation of the case**
**‘Bogged down’**: role of ultimately irrelevant information either explicit (doctor suggesting behaviour for student) or tacit (information load)
**Invisible elephant:** the extent to which students see or do not see feedback that is integrated into the case narrative, but not explicitly labelled
**‘Loss of control’:** students’ perceived loss of control in the case that may or not be related to branching structures
**Pathway decay:** decay in learning, which occurs as a result of losses in time or effort, uncertainty or motivation caused by being allowed to follow different routes
**Contextual dissonance:** factors which clash with previous case assumptions, such as discordant information ‘There were some discrepancies… the age on the GP records is different to when you are given the first stem, there is a 9-year difference, she was born in 1970 in one and 1979, I don't know whether or not that's relevant’FH, Year 4 student, FG1
**False expectations:** students’ false preconceptions about clinical scenarios and professional duties that are detrimental KG: ‘That's the sort of thing you're going to get asked in an exam. Clinically I don't think it's that relevant. I think being able to say, is it likely or is it unlikely ’FH: ‘I don't think we should have to work it out for ourselves…’KG and FH, Year 4 students, FG1 [discussing Bayesian reasoning with immunology laboratory tests]
**Points on the board:** the student's primary focus becomes the assessment and scoring employed during the case
**e-Failure:** a technical failure, the origins of which may be the student, author, VP software, or IT software, hardware and infrastructure
**e-Relationships: how the students form relationships with electronic representations of patients and health care professionals**
**Stereotyping:** stereotyping or making moral professional or personal judgements about case participants FK: ‘She was like “Oh, I have a new partner, I want to start a new family”’ AR: ‘I was thinking, you've left it a bit late’FK and AR, Year 4 students, FG1
**Relationship threshold:** complexity of sustaining more than two relationships in a case (with supervisors, the patient, allied health professionals)
**Hidden agenda: activity of deconstructing the VP and its components either naturally, out of curiosity or to improve performance**
**Assessment subtext:** interpreting the case in the context of the institution or teacher assessment strategies MB: ‘And when you think of it as an exam, you start looking for a style, because everyone has a style in the way they will write a question and answer, and you're trying to link the two up rather than thinking…’JC: ‘What actually should I do?’JC, MB and JC, Year 4 students, FG2,
**Case template subtext:** the student devotes time to exploring real or perceived examiner VP design structure, for interest or to find patterns of assessment ‘It felt to me like essentially you went off for a little tangent for a couple of windows and then it would drop you back onto a common pathway towards to the end of whatever you did’ JW, Year 4 student, FG1
**Handling emotion: students described the process of coping with different emotions during the case, 11 in total:**
**Fairness, Humour, Comfort zone, Uncertainty, Fear, Confidence, Denial, Embarrassment, Pressure, Fatigue and Distraction**‘It seemed quite realistic to me, like kind of both embarrassing and reassuring at the same time. Even though it was simulated, I did feel a bit embarrassed when I was being slightly corrected when I hadn't decided to refer the patient… and so had… “Oh, really you should have referred that… but don't worry I've sent off the referral.”… That's… giving you the feedback in a realistic way, how it probably would happen in real life, and I feel like I'm going to remember that a lot more because of that feeling of embarrassment’AP, Year 2 student, FG5

GP = general practitioner; FG = focus group

**Table 3:** The fourth category from the central phenomenon ‘learning from the VP’, ‘consequences’

Category 4. Consequences:. The results of a student engaging with an individual or series of VPs
**Student change: The impact on knowledge and behaviours in students future practice**
**Real world reasoning**: Incorporating processes taught in the VP that changes clinical practice and approach to patients *“I also liked the multiple choices question part, despite having 10 options for the blood results, what are the three most important ones…..It gets you into the mind-set of not ticking all of the boxes, which in theory you could probably do come August if you wanted to.”* *BN, FG4, Year 4 student, [NOTE: August refers to the month graduates begin work as a qualified doctor]*
**Addressing weakness**: Highlighting areas of knowledge skills or behaviours that are weak, and addressing those areas *“I kind of guessed the first one, and then I realised actually you can work it out… so it highlights your weaknesses I suppose”* AR, FG1, year 4 student
**Individualised experience**: Unique user learning experiences which depends on domains one to three *“I have a different experience from you, again because I didn't look at the score…I stopped and I started doing the modified Schober's test… having that break where I didn't feel like I had to do anything, I was just learning.”* AL, FG1, year four student
**Personal ‘Buy In’ extent to which VP design influences current and future participation of VP cases**
**Learning-realism trade-off**: An apparent trade off between learning and realism when faced with different design properties *“I think the question… is…. do you want learning or realism, because it was better to learn with the linear case, because obviously there's only one way to go with it… if you make the wrong decision…we're not going to learn what the right path necessarily is. Whereas its going to obviously be more realistic… so the second case was more realistic but the first one was a better learning experience.”* MB, FG2, year 4 student
**Future uptake**: The approach to voluntary or compulsory cases in future training

### VP Construction

The first category to emerge from the data was *VP Construction*, which refers to the information encoded into a VP case file by an author. Three sub-categories emerged from the data: *clinical properties*, the clinical elements interwoven into a case; *pedagogic properties*, the educational characteristics of the e-learning case, and *electronic properties*, which include software, electronic usability, data presentation and the student–computer interface. These are defined individually alongside their component codes with examples in Table 1.

### External Preconditions

The second category, of *External Preconditions*, emerged as the baseline characteristics outside the control of the student or authors that influence interaction with the VP. It has three sub-categories. *Student preconditions* describes how a student's clinical and educational experiences, attitudes, knowledge, skills and, in particular, negative experiences with e-learning, play a role in influencing that student's approach to VPs. The sub-category *organisational elements* relates to an institution's approach and policies that relate to curriculum, assessment strategy, learning environment, student appraisal and teaching materials. For example, the component ‘Environmental factors’ describes not only the location, but local factors such as the proximity of students to one another when sitting cases, and the computer hardware used to realise the authored cases. Each of these preconditions appears to influence how a student engages with a VP case. These sub-categories and component codes are defined with examples in Table 1.

### Student–VP Interaction

The third category, *Student–VP Interaction*, is shown in Table 2 and describes the emergent interplay of elements between an individual student and the VP. This is specific to the individual student under the influence of the *External Preconditions* relating to the individual and the organisation, as well as the *VP Construction* (i.e. the first two categories described). This means individual instructional design elements produce different responses in different students. We identified six sub-categories of *Student–VP Interaction* that help to inform instructional design. The *skipping threshold* describes a point at which a student disengages with the case narrative, and the reasons why students skip through without reading or interacting with information presented. An example of how data triangulation has been used to support this phenomenon is shown in Table 4. Here, Student JC, who describes skipping (see quotation in Table 2), is shown to be actually skipping through content in comparison with colleagues and reviewers. We identified three broad reasons for this, which included a student's perspective of ‘efficiency’, the perceived credibility of the case, and the style of learning material. *Mental case building* and *mental case fracturing* describe how cognitive representations of cases are enhanced or sabotaged. *e-Relationships* describes the formation and evolution of relationships between the student and the people represented in the case. Components of *e-relationships* include a ‘relationship threshold’: as the number of characters in the VP rises above three, it becomes difficult for a student to maintain e-relationships with them. *Hidden agenda* reflects student behaviour in terms of the student's treatment of the VP as both a summative assessment with deliberate traps and triggers, and an evaluation subtext in which the student probes for author style, patterns and system vulnerability. *Handling emotions* describes how students deal with the 11 different emotions we identified, which include embarrassment and fear of working with ‘virtual’ colleagues. We present quotations from Student AP (Table 2) which show the embarrassment caused by making a mistake and seem to reflect the realism felt in the case. These emotions appeared to be reinforced by professional stereotypes influenced by a student's experience (*External Preconditions*, Table 1). For example, one view of the behaviour of hospital specialists was that they do not provide positive feedback, exemplified by the comment ‘Consultants don't give out praise’ (Student CB, focus group 2).

**Table 4:** An example of three sources data triangulation

(a) Shows Student JC who had described skipping content in the focus group to have actually skipped content using our data logs. JC spent three-seconds on examination findings, shorter than peers and two reviewers, but performed satisfactorily compared to peers on the case score		

User	Seconds spent on window “Examination of Mrs Begum”	Case score
JC*	**3**	**13**
CB	40	18
JM	25	12
HD	33	16
AA	30	13
MB	52	12
AA	23	15
AM	33	12
Reviewer 1	23	N/A
Reviewer 2	15	N/A

**(b) Shows an example of free text feedback, prior to participating in focus group**		
“I thought the use of lots of information from the history, test results and radiographs made it a very realistic case to work through.”		
WA, Electronic free text comment, prior to FG3, Year 4 student
“It was useful as I had to make the decision on what I would do, but if wrong I was redirected on the right course so I could learn from the case.”
JM, Electronic free text comment, prior to FG2, Year 4 student

**(c) Is a demonstration of CAQDAS supporting the choice of an ‘in vivo’ code, “real life”- the phrase occurs 65 times, and in all focus groups**		
**Focus group**	**Year of training**	**Occurrence of phrase “real life”**
1	4	7
2	4	11
3	4	4
4	4	25
5	2	13
6	2	5
**Total**		**65**

### Consequences

The fourth and final category is *Consequence*s, which describes the result of the student–VP interaction (Table 3). *Consequence*s has two sub-categories: *student change*, which refers to the learning that has occurred, and *preferences and buy-in*, which describes student attitudes to the VP completed and to future VPs. Students describe a unique ‘individualised learning experience’ based on their student–VP interactions shaped by VP construction and preconditions. In alluding to the ‘learning–realism trade-off’, we describe ideal VP characteristics that seemingly cannot both be addressed. An example in Table 3 describes the branching VP case as being more realistic, but as providing an inferior learning experience.

### The model: VP implementation

**Figure 1:**
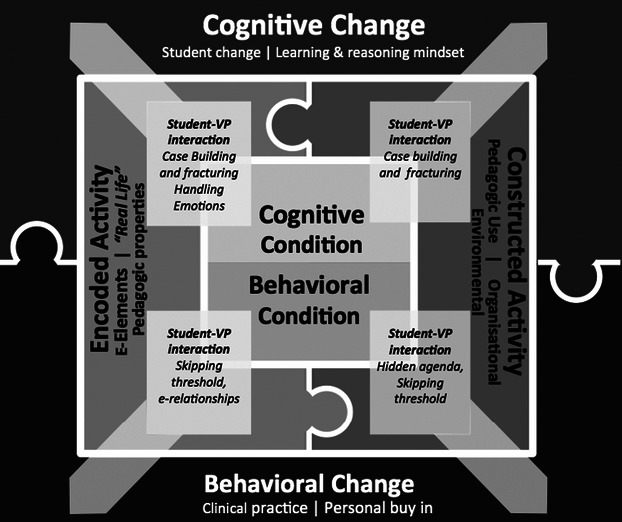
Virtual patient (VP) implementation model. It includes three layers in which *Student–VP Interaction* overlaps the inner two layers and describes how the different ways in which VPs are implemented can influence learning

The model we produced (Fig. 1) is based on and grounded in the data. This model describes and predicts the impact of different design-, institution- and student-related factors on the critical engagement between a student and the VP, the *Student–VP Interaction*. Ultimately, the model predicts the consequences of an individual sitting a VP, co-dependent on the prior factors described. The model has three layers. The inner layer is centred on the student, formed from the ‘cognitive condition’, and ‘behavioural condition’. This describes the student's state prior to interacting with the VP, formed by prior attitudes, knowledge, experiences and skills. The middle layer describes VP delivery as comprising two elements. The first of these is as an ‘encoded object’ and refers to the VP's properties as an e-learning case. These include the electronic, pedagogic and clinical factors described. The second element describes VP delivery as a ‘constructed activity’, which encompasses how an institution delivers teaching using a VP case. ‘Constructed activity’ includes curriculum, pedagogic context and the environment in which VPs are delivered. Overlapping the inner two layers in our model is the *Student–VP Interaction*, which describes the critical behavioural interactions dependent on the student, and encoded and constructed activity. The outer layer represents the product of the interaction, learning, as ‘cognitive change’ and ‘behavioural change’.

## Discussion

We have explored a number of design features in VP cases created to established technical standards in order to build theory in this important and under-researched area.^6^ This original research has produced a model (Fig. 1) that explains how and why different VP design features influence learning. We describe a series of six new phenomena within the *Student–VP Interaction* that help to give a deeper understanding of how and why a student engages with and learns from a VP. Clinicians may or may not be aware of these interactions and the importance and impact of encoded and constructed activity. Thus, they may not be either consciously or unconsciously incorporating design variables to accommodate these factors.

The multiple design variables used to author the VPs are part of the encoded activity. Some design properties produced a mixture of positive and negative effects. These included: case pathway choices, such as branching versus linear case narratives; some structured approaches to clinical reasoning using the evidence available, and data quantity and presentation (such as the use of additional information, which added to ‘clinical inertia’, and scoring counters). Some elements intended to promote sound clinical reasoning appeared at times to frustrate students, such as prompting ‘non e-tasks’ and some case presentation ‘e-elements’, such as the scoring counter. As evidence emerges for these approaches in clinical medicine, careful consideration to their application can be informed by our model. This could include incorporating structured reasoning as an electronic task, and limiting the intrusion of these processes into the realism of a case.

For example, the scoring counter helped some students in ‘learning and assessment focus’, but produced negative behaviours including that of lowering the *skipping threshold* in some students seeking to obtain ‘points on the board’ (Table 2).

The model highlights a trade-off between certain design possibilities and student behaviour and the consequences of these for learning. Our model prompts authors to consider the opportunities and constraints of ‘constructed activity’, such as those imposed by technical facilities, curricular integration and participants, and the attributes of the VP audience, the ‘student preconditions’. Each apparently desirable design feature, such as complex branching with realistic ‘clinical inertia’, has positive and negative effects on students. The model predicts that using a branched case design potentially makes cases more realistic, but is associated with increased complexity and a higher frequency of and more types of ‘e-inertia’ and errors (Table 1). The resulting effects this appears to have on *Student–VP Interaction* in terms of the *skipping threshold*, ‘cognitive dissonance’ and ‘case fracturing’ (Table 2) are complex and different for each student. These cannot be abstracted from this model to a general recommendation supporting or opposing a design variable such as ‘branching’, but can only help to provide context for that decision based on the other components of the model (the students, constructed activity).

We did find general consensus in favour of and against some design properties. ‘e-Signposting’ and the use of ‘key feature problems’ and Bayesian reasoning questions were helpful; the latter two represent existing validated measurements of clinical reasoning skills.^28^ The item difficulty in these questions produced interesting phenomena such as that of ‘perceived e-error’. Poorly received design properties included problems with the narrative (‘relationship saturation’), information presentation (‘scroll scroll scroll’, an ‘e-property) (Table 1), and feedback (the ‘invisible elephant’, a component of ‘case fracturing’) (Table 2).

This was an innovative research project in two key areas: the research methodology, and its transparent reporting. Firstly, our methodology utilised available computer-assisted technology to identify, describe and triangulate new findings. These data were used to help understand individual interactions with VPs, and neither used nor planned for quantitative analysis. To our knowledge, this is the first VP research study to explicitly construct VPs to established software standards, with the single purpose of researching important design properties such as branching cases. Our second-by-second individual student data logs and self-reported evaluations (Table 4) allow the triangulation and exploration of theoretical constructs. These have contributed to the descriptions of new and interesting phenomena in *Student–VP* Interaction, such as the *skipping threshold*, and link described with actual behaviour (Table 4), facilitating comparison with observed clinical practice. We consider this skipping to be in many ways analogous to mind wandering.^29^ Secondly, our open transparent representation of research includes unprecedented detail in both the descriptions and schematics of the VPs used (Table S1 and Fig. S1) and the XML case files (details available from the authors), which have not been included in recent research into VPs.

This work builds on the existing literature on VPs, and emphasises that what an author encodes is only one of many variables contributing to educational impact. This model adds to research on design principles,^2^,^6^,^12^ practical authoring advice,^30^,^31^ curricular integration,^32^ the theoretical principles behind the VP,^33^ the importance of the environment,^34^ and the difficulties inherent in sharing and repurposing VPs.^35^ Our work supports 10 general authoring recommendations produced from a thematic analysis of VPs in focus groups,^12^ and provides a framework within which any characteristic, such as authenticity, can be considered within our model.

### Limitations

Some design elements lay outside the scope of this investigation. We chose not to study video or audio files as design properties for reasons of cost, technological limitations for distribution, and lack of flexibility for updating and repurposing. We did not study natural language input or free-text questioning. Although equivalents have been used in some VP research,^12^ they are unavailable in most open-source and some commercial VP players^31^ and to our knowledge do not have an open technical standard equivalent.^17^ We cannot exclude the possibility that the presentation effects of the software interface we used, DecisionSim, may have an impact. We did not investigate a number of areas, such as the use of VPs as part of group work,^6^,^36^ different curricular integration strategies,^24^,^32^ novel approaches to teaching clinical reasoning such as the ‘think-aloud’ approach,^37^,^38^ or address specific situations such as cognitive bias.^39^ That said, most of these approaches describe either how an institution deploys a VP, and thus might fit into our model as ‘constructed activity’; the others reflect elements of the encoded activity.

We have attempted to address as far as possible the problems associated with bias, reflexivity, observer effects and researcher preconceptions using a number of methods. When considering reflexivity, the impact of the researchers’ position and perspectives, we acknowledge that we consider that VP design is important and this stance may potentially result in an inductive bias and influence the participants, analysis and conclusions. We argue that our subjection of the research protocol to an iterative process of both internal and external peer review has helped to prevent this, as have regular institution review board meetings at which analysis was presented.^15^,^16^,^40^ Other grounded theorists would question the validity of many of these approaches and, for example, may not agree with our use of multiple data sources and triangulation.^16^ All of these approaches would be supported by the school of grounded theory to which we subscribe.^15^ Participants were part of a study in one institution, which raises the possibility of observer effects.^41^ To minimise these, the research study was completed in a familiar learning environment. Our research may not be transferable to other institutions and health care professional groups. The extent to which different groups rely more or less on VP cases in their curricula may also influence this.^7^ We have observed that our themes did recur across year groups, in students who had and had not previously used VPs. We did not seek ‘respondent validation’ for our theory; we are in agreement with the literature that proposes this represents another data source for analysis rather than a form of theory validation.^42^,^43^

### Practical implications for medical education

We have produced a model that has practical use for stakeholders who may be authoring or commissioning VPs. The model describes how VPs produce different learning experiences in a framework that incorporates students, authors, technical and software elements, institutions and environments. Developers can consider which design features and electronic presentation to adopt, the ‘encoded activity’, and how they should be delivered, the ‘constructed activity’. In a consideration of these and ‘student preconditions’, which refers to, for example, how electronic prejudices are formed, and how they all influence types of *Student–VP Interaction*, the present framework may help to elucidate how a VP might be developed for any given topic. Researchers can also use the framework to consider how to plan and report new research to help inform VP adoption, repurposing and sharing, against a backdrop of challenges to the resourcing of education for medical and allied health professionals.^44^ For example, a senior allied health professional teaching on a national Masters-level course could consider the impact of student preconditions and how existing VP cases might be adapted or repurposed (encoded activity) within the environment and resources (constructed activity) to achieve the objectives inherent in the use of VPs (cognitive and behavioural change).

We hope other educators, researchers and institutions will incorporate this model when developing and researching VPs, and call for researchers to follow transparent reporting of VP design properties. We hope this work will help to inform the interpretation of further research we are conducting into measuring the impact of different VP designs.^45^

## SUPPORTING INFORMATION

**Table S1.** Design of cases.

**Appendix S1.** Virtual Patient XML case files used in the research.
