# Supplementary material for: Virtual patient design: exploring what works and why. A grounded theory study
Source: Med Educ. 2013 May 12;47(6):595–606. doi: 10.1111/medu.12151 (PMC3677415; doi:10.1111/medu.12151)

| S1. Supplementary Material. Design of Cases | | | |
| --- | --- | --- | --- |
|  | *Case 1* | *Case 2* | |
| **Overview** | | | |
| *Case Narrative* | 31-year-old Caucasian male, back pain, mechanical evolving to inflammatory sero-negative arthritis with knee effusion | 27-year-old Asian female, arthralgia and compression neuropathy evolving to inflammatory arthritis (rheumatoid arthritis). | |
| *Final Diagnosis* | Ankylosing Spondylitis, inflammatory knee arthritis | Rheumatoid Arthritis, carpal tunnel syndrome | |
| *Role of student sitting VP* | Newly qualified doctor | Newly qualified doctor | |
| *Target Audience* | Clinical Medical Students | Clinical Medical Students | |
| *Setting* | Primary Case | Secondary Care | |
| *Number of characters authored into case.* | 3- Patient, General Practitioner, Rheumatologist | 4- Patient, Orthopaedic surgeon, Physiotherapist, Rheumatologist | |
| *Time period Simulated* | Months | Months | |
| *Principle Author* | Author 1, Education Research Fellow | Author 1, Education Research Fellow | |
| *Authoring platform* | DecisionSim V2.0 | DecisionSim V2.0 | |
| *Quality control* | Peer review, piloting | Peer review, piloting | |
| **Case Properties** | | | |
| *Total Number of nodes (steps)* | 49 | 68 | |
| *Case Type* | Overall Linear, see S2 for further details | Overall Branched, see S2 for further details | |
| *Number of Branches* | 1 | 3 | |
| *Substantial routes through VP* | 1 | 27 (3^^^3) | |
| *Choices at each branching point* | 3 (users redirected down linear path) | 3 | |
| *Minimal steps to complete VP* | 28 | 28 | |
| *Node types* | Question, branching, enquiry | Question, branching, enquiry | |
| *Time allocated by author* | 30 minutes | 30 minutes | |
| *Number of ‘Wheel and spoke hubs** | 3 | 2 | |
| **Supporting Media** | | | |
| *Number of images in the case* | 24 | | 16 |
| *Supporting images: Clinical* | Physical Examination; Blood tests; GALS Screen, Annotated and normal Radiographs; Authentic reports (Radiography , microbiology, lab) | | Physical Examination; Observation Chart; Blood tests; GALS Screen; Annotated and normal Radiographs; Patient HAQ ** |
| *Supporting images: Miscellaneous* | Pictures of participants, environment, letterheads for correspondence, electronic results screens | | Pictures of Patient, environment, letterheads for correspondence, electronic results screens |
| *Audio files* | No | | No |
| *Video files* | No | | No |
| **Instructional Design Features Studied** | | | |
| *Structured promotion reasoning* | No | Yes | |
| *History Taking: Enquiry chosen by student* | Yes | Yes | |
| *History Taking: Information pre*  *Given in authentic health care records* | Variable through case | Variable through case | |
| *Investigation results: authentic presented* | Yes, some | Yes, some | |
| *Investigation results: text only presented* | Yes, some | Yes, some | |
| *Extra teaching resources within case* | Yes | No | |
| *Extra teaching resources following case* | No | Yes | |
| *Deliberate Errors included* | No | Yes | |
| *Natural Language entry* | No | No | |
| *Visual Signposting* | Majority | Majority | |
| *Opportunity to question Peer* | No | Yes | |
| **Common design Features** | | | |
| **In Case Questions and Assessment** | | | |
| *Bayes Reasoning, (n=1), Key feature problem style (10), Clinical decisions (3)* | | | |
| **Feedback in the cases** | | | |
| Both given immediately following decisions and delayed to be presented authentically in case evolution.  Includes why answers both right and wrong. Feedback given explicitly flagged as feedback, and tacit. | | | |
| **Variable design features in case 1 or case 2** | | | |
| Visible Score (present vs. absent), Navigation (open navigation vs. closed), timer (present vs. absent) | | | |
| **Wheel and spoke refers to points where students an carry out different actions before carrying on down the linear pathway, for further information see* *Huwendiek S, et al. Towards a typology of virtual patients. Med Teach. 2009 Aug;31(8):743-8; **HAQ refers to the Health Assessment Questionnaire, a self reported patient questionnaire on wellbeing.* | | | |

## S2 Schematic of Layout of the Cases


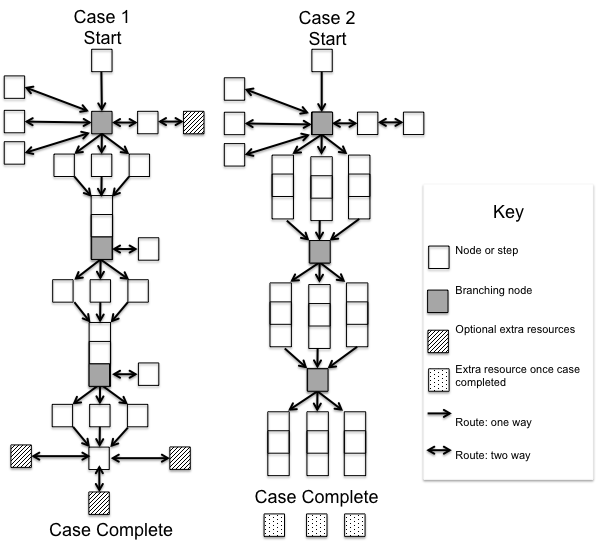

Supplement: Supplementary file 1 [file medu0047-0595-SD1.docx]
