# Supplementary material for: Virtual patient design: exploring what works and why. A grounded theory study
Source: Med Educ. 2013 May 12;47(6):595–606. doi: 10.1111/medu.12151 (PMC3677415; doi:10.1111/medu.12151)
Supplement: Supplementary file 2 [file medu0047-0595-SD2.doc]

**Supplementary information S3.**

| **Contents** | **Title** | **Page** |
| --- | --- | --- |
| **S3 (a)** | **Focus group introductory statement** | **1** |
| **S3 (b)** | **Funnelled questioning route** | **2** |
| **S3 (c)** | **Participant information sheet** | **3** |
| **S3 (d)** | **Written consent form** | **4** |

**S3 (a) Focus group introductory statement (redacted**

**[Introduction, to be read verbatim by facilitator]**

Good morning and thank you for attending this focus group session on Virtual Patients. My name is X, a research student here at Warwick University. This is Y, a facilitator.

You have been chosen because you are medical students studying here at Warwick, and we are interested in your opinions on the virtual patient cases you have been given to work through. Thanks for completing the virtual patient cases.

Although I will be moderating the discussion today, I am only interested in your own contributions, and I won’t be responding to your contributions except for clarification. We are interested in your opinions and experiences of the Virtual Patients, in particular how the cases are organised and designed, rather than the content itself.

Remember that there are no right and wrong answers, only opinions. The literature on what works best with virtual patients is unclear, which is why we are conducting these focus groups.

You may have different views to other students, and that’s partly what we are interested in. We are keen to get everyone’s view, and because of this you can of course disagree with each other.

We would like to record this session as was initially discussed with you. This will be secure and used only for the research study. There and as we are recording the discussion I would ask it only one person at a time speaks, for the property of recording.

The discussion will remain anonymous: no one will be identified by name in the research. As we have discussed your participation is voluntary. The focus group will last for about one hour. This research project has been through an ethical review process and Warwick University’s Biomedical Research and Ethics Committee, and you have already seen the information sheet. As previous the discussion is voluntary, and you are free to leave at any time. Please help yourself to a drink, and does anyone have any questions before we start?

**S3 (b) Funnelled questioning route (v1.4)**

**DURATOIN 1 HOUR (00:00 TO 60:00)**

**OPENING QUESTION. Response from every student needed [00:00- 05:00]**

**[1]** Welcome to the focus group. Perhaps we can all go round and introduce ourselves, perhaps your name, and a tell me interesting you have seen in your last clinical rotation.

**INTRODUCTARY QUESTION [05:00- 10:00]**

**[2]** The two cases you have sat are “Virtual patients”, and teachers think use for them will be to in different ways. Have any of you used anything like this before?

*[optional] Do you have any comments general on the cases?*

*What did if anything did you learn from them?*

**TRANSITION QUESTION [15:00]**

**[3]**Like many areas of education, there’s little evidence on how best to design and structure virtual patients cases to teach a certain area and help students learn. Do you think it is important about how these virtual patient cases are designed?

**KEY QUESTIONS [15:00- 50:00- 5 MINUTES PER QUESTION, 10 MINUTES LEEWAY]**

**[4]** In each VP case your choices lead you down a certain path*way*. Did you notice a difference between how this worked in the two cases?

*[Optional] If so, do you think it made much difference?*

*If you noticed this, How and why did you think it affected how good the case was?*

*[Optional] One of the cases actually had quite linear path, despite the different choices you had, whereas in the other case you could go down a number of different routes. Reflecting on the cases, how, if at all, did this affect you when completing the case?*

**[5]** We would like the cases to help you make and weight up clinical decisions: thinking about how you make a diagnosis or choose a treatment. Think back to the cases: in one, you were regularly prompted from the outset to think about, reflect on, sift through, and modify the possibilities. How do you think this approach worked, and for example would you change or improve the prompting in the case?

**[6]** In the cases, you were asked to answer lots of different styles of questions, and make decisions. Do you think how the questions were constructed was OK?

**[7]** You had a number of different types of feedback through the cases. Were there any aspects of the feedback that you liked or did not like, and why*?*

**[8]** There are lots of ways that these cases can be put together as you have started to see. With this in mind, was there anything else, good or bad that we haven’t discussed that you thought was important?

**CLOSE: Response from every student [50:00]**

**[9]** Finally, I would like to ask each of you individually a closing question. I would like each of you to pick out one design feature of the VP that you liked, and why, and one improvement you would make to either of the cases. So perhaps “***first student”*** you could start:

**S3(c) Participant information sheet [redacted]**


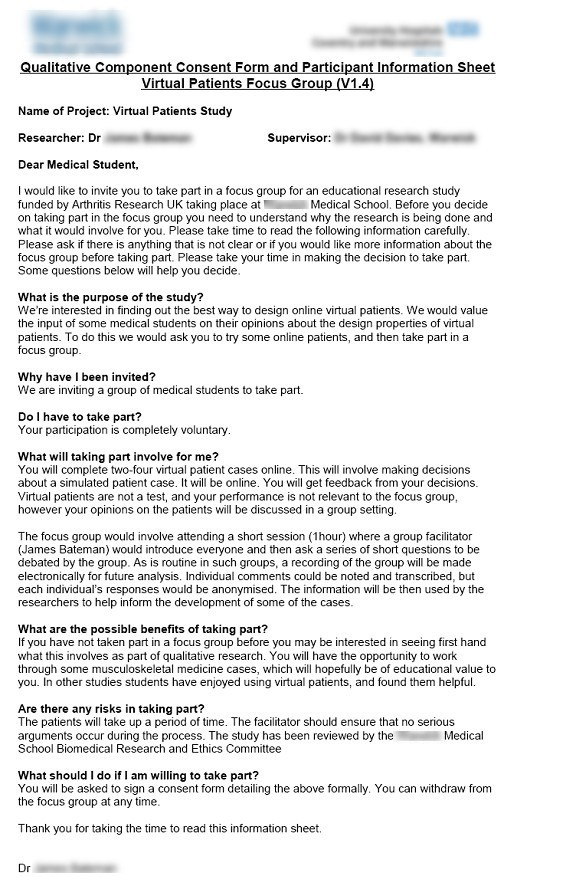


S3(d) Written Consent form


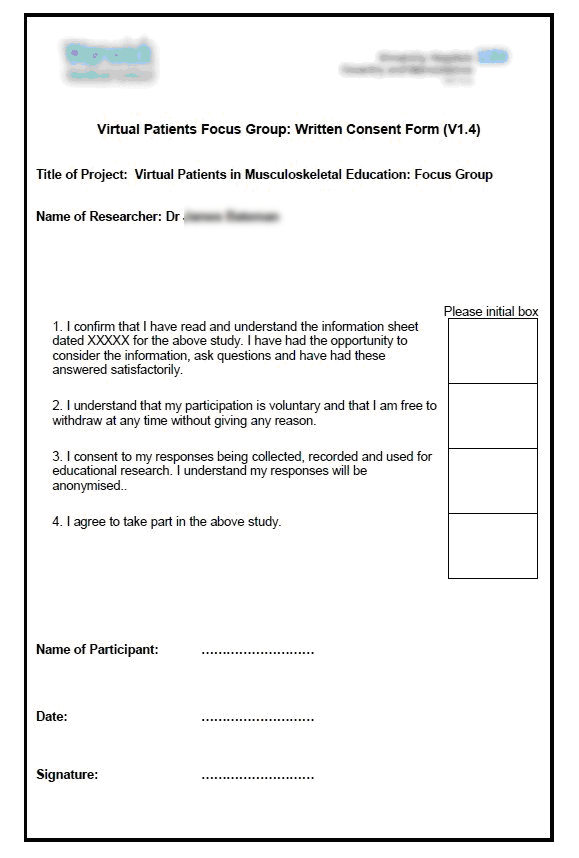


**Version XXXXX Date XXXXXX**
